# Efficacy of sodium butyrate adjunct therapy in shigellosis: a randomized, double-blind, placebo-controlled clinical trial

**DOI:** 10.1186/1471-2334-12-111

**Published:** 2012-05-10

**Authors:** Rubhana Raqib, Protim Sarker, Akhirunnesa Mily, Nur Haque Alam, Abu Saleh Mohammed Arifuzzaman, Rokeya Sultana Rekha, Jan Andersson, Gudmundur H Gudmundsson, Alejandro Cravioto, Birgitta Agerberth

**Affiliations:** 1 International Centre for Diarrheal Disease Research, Dhaka, Bangladesh; 2 Department of Medical Biochemistry and Biophysics, Karolinska Institutet, Stockholm, Sweden; 3 Center for Infectious Medicine, Department of Medicine, Karolinska University Hospital Huddinge, Stockholm, Sweden; 4 Institute of Biology, University of Iceland, Reykjavik, Iceland; 5 Nutritional Biochemistry Laboratory, Laboratory Sciences Division, International Centre for Diarrheal Disease Research, Bangladesh (icddr,b), Mohakhali, Dhaka, 1212, Bangladesh

**Keywords:** Short chain fatty acids, Butyrate, Shigellosis, Innate immunity, Antimicrobial peptides, Cathelicidin, LL-37, Inflammation, Pro-inflammatory cytokines, Rectal mucosa

## Abstract

**Background:**

Treatment of shigellosis in rabbits with butyrate reduces clinical severity and counteracts the downregulation of cathelicidin (CAP-18) in the large intestinal epithelia. Here, we aimed to evaluate whether butyrate can be used as an adjunct to antibiotics in the treatment of shigellosis in patients.

**Methods:**

A randomized, double-blind, placebo-controlled, parallel-group designed clinical trial was conducted. Eighty adult patients with shigellosis were randomized to either the Intervention group (butyrate, n = 40) or the Placebo group (normal saline, n = 40). The Intervention group was given an enema containing sodium butyrate (80 mM), twice daily for 3 days, while the Placebo group received the same dose of normal saline. The primary endpoint of the trial was to assess the efficacy of butyrate in improving clinical, endoscopic and histological features of shigellosis. The secondary endpoint was to study the effect of butyrate on the induction of antimicrobial peptides in the rectum. Clinical outcomes were assessed and concentrations of antimicrobial peptides (LL-37, human beta defensin1 [HBD-1] and human beta defensin 3 [HBD-3]) and pro-inflammatory cytokines (interleukin-1β [IL-1β] and interleukin-8 [IL-8]) were measured in the stool. Sigmoidoscopic and histopathological analyses, and immunostaining of LL-37 in the rectal mucosa were performed in a subgroup of patients.

**Results:**

Compared with placebo, butyrate therapy led to the early reduction of macrophages, pus cells, IL-8 and IL-1β in the stool and improvement in rectal histopathology. Butyrate treatment induced LL-37 expression in the rectal epithelia. Stool concentration of LL-37 remained significantly higher in the Intervention group on days 4 and 7.

**Conclusion:**

Adjunct therapy with butyrate during shigellosis led to early reduction of inflammation and enhanced LL-37 expression in the rectal epithelia with prolonged release of LL-37 in the stool.

**Trial Registration:**

ClinicalTrials.gov, NCT00800930.

## Background

Shigellosis continues to be a major health burden with an annual incidence rate of 125 million cases in Asia, although the fatality rate has decreased substantially over the last two decades [1]. Invasion of the colonic mucosa by *Shigella* leads to inflammation-mediated destruction of the mucosal barrier. The resultant manifestations are the passage of bloody mucoid loose stools, abdominal cramps, rectal tenesmus and fever [2]. The management of shigellosis depends on antibiotics, but the emergence of antibiotic-resistant strains is limiting their effectiveness. Moreover, it is often the case that antibiotic treatment cannot resolve the chronic inflammatory response of shigellosis [3,4]. Furthermore, the use of antibiotics can have systemic side-effects and disturb the balance of normal flora, exacerbating the disease or making the host prone to other opportunistic pathogens [5]. Therefore, new drugs need to be developed that may act alone or as adjunct to antibiotic therapy.

Antimicrobial peptides (AMPs) are front-line components of innate immunity in multicellular organisms [6]. These peptides constitute an antimicrobial arsenal against a wide range of pathogens at the host-microbe interfaces. Our group and others have previously shown that *Shigella* spp downregulates the expression of human cathelicidin LL-37 and beta defensins in colonic epithelial cells, one of several mechanisms employed by this pathogen to evade host defenses [7,8].

Short chain fatty acids (SCFAs), primarily acetate, propionate and butyrate are bacterial fermentation products of undigested dietary carbohydrates in the colon. SCFAs, principally butyrate, supply energy and exert various effects on colonocytes, influencing colonic health [9]. Rabbani *et al.* first demonstrated that a mixture of SCFAs can improve the clinicopathologic and bacteriologic features of experimental shigellosis in rabbits [10]. Later, we showed that oral butyrate treatment of rabbits leads to clinical recovery and reduced *Shigella* count in the stool [11]. In addition, *Shigella-*mediated downregulation of cathelicidin CAP-18 in the large intestinal epithelia of rabbits was counteracted by butyrate treatment [11]. In a randomized clinical trial, ingestion of green banana, which induces luminal SCFA production, reduced clinical severity of childhood shigellosis [12]. However, to our knowledge, no study has been carried out to evaluate the efficacy of butyrate therapy on recovery following shigellosis in humans. Therefore, in this study, we aimed to assess the potential of butyrate as an adjunct to antibiotic therapy in adult shigellosis in terms of clinical, endoscopic and histopathological recovery. The effect of butyrate on the expression of antimicrobial peptides and pro-inflammatory cytokines was also investigated.

## Methods

This study is reported according to CONSORT (Consolidated Standards of Reporting Trials) guidelines.

### Study design

A double-blind, placebo-controlled, parallel-group designed, equally randomized (1:1) clinical trial was conducted in Bangladesh from January 2005 to January 2009. The Trial Registration number is ClinicalTrials.gov, NCT00800930.

### Ethics statement

The trial was conducted in accordance with the declaration of Helsinki. The study protocol was approved by the Ethical Review Committee of the International Centre for Diarrheal Disease Research, Bangladesh (icddr,b) and by the Directorate General of Drug Administration (DGDA) of the Government of the People’s Republic of Bangladesh. Written informed consent was obtained from all eligible participants before participation.

### Intervention compound

Sodium butyrate was purchased from Merck Schuchardt OHG, Hohenbrun, Germany and the analysis for pharmaceutical grade was performed by Apoteket Production Laboratory, Stockholm, Sweden.

### Selection of patients, study settings and locations

Adult (18–55 years) patients of both sexes having occult blood and mucus in their stool and with a history of 0–4 days of diarrhea were selected as presumptive cases of shigellosis in the outpatient clinic of the Dhaka Hospital and the Matlab Hospital of icddr,b. The Dhaka Hospital serves the metropolitan district of Dhaka and its surrounding areas, while the Matlab Hospital provides health care in a rural setting.

### Clinical management

Following selection, standard clinical history was taken and physical examinations were performed. All patients were given pivmecillinam (400 mg, every 8 hours for 5 days) as empirical therapy. If required, oral or intravenous rehydration was given to patients during hospitalization. The patients were kept in the study ward for 4 days to administer enema and were released on the 5^th^ day. If the diarrhea did not subside by 5^th^ day, they were kept in the study ward for additional days until diarrhea resolved. Standard treatment was maintained, even if the patients were no longer in the study. All patients received the usual hospital food three times a day.

### Inclusion and exclusion criteria for enrollment

Patients aged 18–55 years with 0–4 days duration of diarrhea and with culture-confirmed *Shigella* spp (all *Shigella* spp.) in their stool were eligible for the study, if they did not meet any exclusion criteria. The exclusion criteria were: (1) treatment with antimicrobial agents before attending the icddr,b hospital; and (2) presence of clinical symptoms of other concomitant infections such as chronic respiratory infections, other concomitant gastrointestinal infections.

### Outcome measures

The primary endpoint of the trial was to assess the efficacy of sodium butyrate enema in improving clinical, endoscopic and histological features of shigellosis. Clinical scoring was set to evaluate the clinical status (given below). Reduction in clinical scores indicated improvement. The levels of pro-inflammatory cytokines, interleukin-8 (IL-8) and interleukin-1β (IL-1β) were assessed in the stool of all patients to support endoscopic and histopathological features of rectal inflammation, which were analyzed in a subgroup of patients.

The secondary endpoint was the induction of endogenous AMPs in the rectum through butyrate treatment. The release of LL-37, human beta defensin 1 (HBD-1) and human beta defensin 3 (HBD-3) in the stool, and the expression of LL-37 in the rectal mucosa were evaluated.

### Sample size calculation

Sample size estimation was based on the assumption of a 30% clinical improvement in the Intervention group over the Placebo group at a 5% significance level with 80% statistical power. The estimated sample size was 38 in each group. Accounting for 5% loss to follow-up or dropouts, the sample size was finalized at 40 cases per group.

### Randomization, allocation concealing, blinding, implementation and intervention

Patients were randomized with a 1:1 allocation ratio using a simple randomization procedure (computer generated list of random numbers prepared by an independent person not involved in the study) to either the Intervention group (butyrate, n = 40) or the Placebo group (normal saline, n = 40). An independent pharmacist dispensed either sodium butyrate or placebo into bottles and consecutively numbered the bottles for each patient according to the randomization list. Enrollment of patients to the study ward was carried out by the nursing staff on duty. The assignment of patients to the Intervention or Placebo group was concealed to both the investigators and patients by enclosing the assignment card that contained the code number for each patient in a sealed envelope. The envelope was opened only at the time of allocating patients to the study by the responsible physician. After enrollment, the Intervention group received 80 mM sodium butyrate isotonic enema, every 12 hours for 72 hours. Sodium butyrate was dissolved in water and osmolarity of the enema solution was adjusted to 295 mOsm/L with sterile nonpyrogenic normal saline. The Placebo group received the same dose of normal saline enema (308 mOsm/L). The enema solution was administered over 7–8 minutes after which the patients were kept in a supine position for another 60 minutes. If the patients could not retain the enema for 30 minutes due to defecation, they were given a 2^nd^ dose of enema.

### Clinical investigations and follow-up visits

Patients were observed for clinical outcomes for 4 consecutive days starting from the enrollment day (day 1). Each clinical parameter was given grading scores: duration of diarrhea: 1 = 1-2 days, 2 = 3 days, 3 = 4 days; stool frequency: 1 = 1-3 times, 2 = 4-7 times, 3 ≥8 times; stool output (g): 1 = 0-76, 2 = 77-190, 3 = 191-382, 4 ≥383; stool consistency: 1 = formed, 2 = soft, 3 = watery; body temperature (°C): 1 = 36.1-37.4, 2 ≥37.5; for vomiting, tenesmus, dehydration, abdominal pain, anorexia and mucus in stool: 1 = absent, 2 = present. Scores for each parameter were combined to set an overall clinical score. Frequency of red blood cells (RBC), pus cells and macrophages in the stool was observed by routine microscopic examinations (RME). The grading for RBC was: 1 = 0, 2 = 1-10, 3 = 11-20, 4 = 21-50 and 5 >50; for pus cells: 1 = 0-10, 2 = 11-20, 3 = 21-50 and 4 >50; for macrophages: 1 = 0, 2 = 1-5, 3 = 6-10 and 4 > 10. If diarrhea subsided, patients were released from the hospital on 5^th^ day and asked to return for a follow up visit on day 7.

Sigmoidoscopic (Olympus Tokyo, Japan) examination was performed in patients, enrolled only in Dhaka Hospital (Intervention group, n = 15 and Placebo group, n = 11) on day 1 and day 7 to monitor inflammation in the rectal mucosa. Inflammation was graded as mild, moderate or severe based on the modified Baron score [13]. One patient in the Placebo group declined to undergo sigmoidoscopic examination on day 7. However, follow-up stools were collected from that patient. Therefore, that patient was excluded from the sigmoidoscopic and subsequent histologic and immunohistochemical analyses. Due to lack of endoscopic facilities in the Matlab Hospital, patients in Matlab (n = 49) did not undergo sigmoidoscopic examination. The patients who underwent sigmoidoscopy are referred hereafter as a subgroup.

### Specimen collection and processing

Stool specimens were collected from each patient on the day of enrollment (day 1), on each of the following 3 days and on day 7. Stool samples were diluted 10-fold with 60% acetonitrile in 1% aqueous trifluoroacetic acid (TFA) and extracted overnight at 4°C. The extracts were centrifuged and supernatants were passed through a 0.45 μm filter, aliqouted, lyophilized and stored at −20°C.

Rectal biopsy samples, 10 to 12 cm from the anus, were collected from patients enrolled in the Dhaka Hospital on days 1 and 7. At each time point, 3 biopsy samples were obtained, fixed in buffered formalin, embedded in paraffin and cut into 3 μm thick sections by a microtome (RM 2055, Leica, Heidelberg, Germany). The sections were mounted on vectabond-coated glass slides (Superfrost/plus, Menzel-Glaser, Germany), dried overnight at 37°C and kept at room temperature.

Blood was collected on days 1 and 4 for toxicity assessments.

### Bacterial count in stool

Bacterial load in the stool was quantified by plating serial dilutions of stool onto MacConkey agar plates with colonies being counted following overnight incubation at 37°C. The results were expressed as colony forming units (CFU) per gram of stool.

### Enzyme linked immunosorbent assay (ELISA)

LL-37, HBD-1, HBD-3, IL-8 and IL-1β were measured in stool extracts by sandwich ELISA. Lyophilized stool extracts were dissolved in Tris-buffer saline (TBS) for LL-37 or in appropriate dilution buffer as recommended by the manufacturer for HBD-1, HBD-3 (Alpha diagnostic, Texas, USA), IL-8 and IL-1β (BD Biosciences Pharmingen, California, USA).

For LL-37 concentration, an in-house method was used. Microtiter plates (96-well, black; Nunc, Roskidle, Denmark) were coated with monoclonal anti-LL-37 (produced in our laboratory). After blocking nonspecific sites, standards (synthetic LL-37 peptide [Innovagen AB, Lund, Sweden]) and stool samples were added in duplicate and incubated overnight at 4°C. Plates were then incubated with biotinylated polyclonal LL-37 (Innovagen AB) and streptavidin-alkaline phosphatase (Millipore, California, USA) for 2 hours each at room temperature. Finally, substrate 4-methylumbelliferyl phosphate (4-MUP) (Invitrogen, Leiden, The Netherlands) was added to produce a fluorescent end product. The fluorescent intensity was measured by Infinite 200 spectrophotometer (Tecan, Männedorf, Switzerland) at an excitation wavelength of 360 nm and an emission wavelength of 450 nm. The LL-37 concentration in stool extracts was then extrapolated from the standard curve.

ELISA for HBD-1, HBD-3, IL-8 and IL-1β was carried out according to manufacturer’s instructions.

### Histology and immunohistochemistry

Paraffin embedded rectal biopsy sections were deparaffinized, stained with hematoxylin and eosin and examined by a pathologist. Histological grading of inflammation as mild, moderate or severe was done according to the criteria described earlier [14].

For immunohistochemical detection of LL-37, deparaffinized sections were microwave-treated in retrieval buffer (Dako, Glostrup, Denmark) and endogenous peroxidase activity was quenched by hydrogen per-oxide (H_2_O_2)._ The sections were then incubated overnight with rabbit polyclonal LL-37 antibody (3 μg/ml) (Innovagen AB), followed by sequential incubation with biotinylated goat anti-rabbit IgG (Dako) and avidin-biotin horseradish peroxidase complex (Dako), each for 1 hour. The color reaction (brown) was developed by adding diaminobenzidine (Dako) as the substrate for peroxidase enzyme. To control for specific staining, synthetic LL-37 peptide was incubated overnight at 4°C at a 20-fold higher concentration with the LL-37 antibody, and the mixture was used for immunostaining.

### Image analyses

*In situ* immunohistochemical staining of LL-37 was analyzed by the image analysis system Quantimate Q550 (Leica, Wetzlar, Germany) according to Cunnane G *et al.*[15]. The epithelial and non-epithelial areas of rectal mucosa were separately assessed for the quantification of LL-37 staining in each tissue section at 400x magnification and the results were given as ACIA (Acquired Computerized Image Analysis) score. Since there were erosions of surface epithelium (SE) at multiple locations, ACIA scores of LL-37 in SE were expressed in terms of ACIA score per unit SE length.

### Biosafety evaluation of treatment

For biosafety evaluation, the serum levels of biomarkers for kidney (urea and creatinine) or liver (alanine transaminase and γ-glutamyl transferase) toxicity were assessed.

### Statistical analyses

Statistical analyses were performed using the statistical software packages SigmaStat (version 3.1; Systat Software Inc., Point Richmond, CA, USA) and SPSS for Windows (release 17; SPSS Inc, Chicago, Illinois, USA). Data were expressed as number of patients, n (% of patients) for categorical variables, and as mean with standard deviation or median with 25–75 percentiles for quantitative variables. Changes of categorical variables over time in two groups were compared using Chi-square test. Quantitative data were transformed (e.g. natural log or log) when not normally distributed, and analyses were performed on the transformed variables. Two-way repeated measures ANOVA was performed to determine significant interaction between butyrate and placebo therapy on different days, and when interaction was significant, the Holm-Sidak post-hoc comparison procedure was used to compare the effects of butyrate therapy on outcome measures. The overall significance level was set at *P* < 0.05.

Effect sizes (ES) of butyrate and placebo adjunct therapy were estimated. ES can be interpreted in terms of the average percentile standing of the average experimental (Intervention) group relative to the average control (Placebo) group or in terms of the percent of non-overlap of the Intervention group's scores with those of the Placebo group. An ES of 0.0 to 0.2 indicates that the mean of the Intervention group is between 50 and 58 percentiles of the Placebo group, and the score distribution for the Intervention group overlaps almost completely with the score distribution for the Placebo group with 0% to 14.7% of non-overlap (small). An ES between 0.6 and 0.8 indicates that the mean of the Intervention group is between 73 and 79 percentiles (large) with 38.2% to 47.4% of non-overlap, respectively.

## Results

### Demography

**Figure 1:**
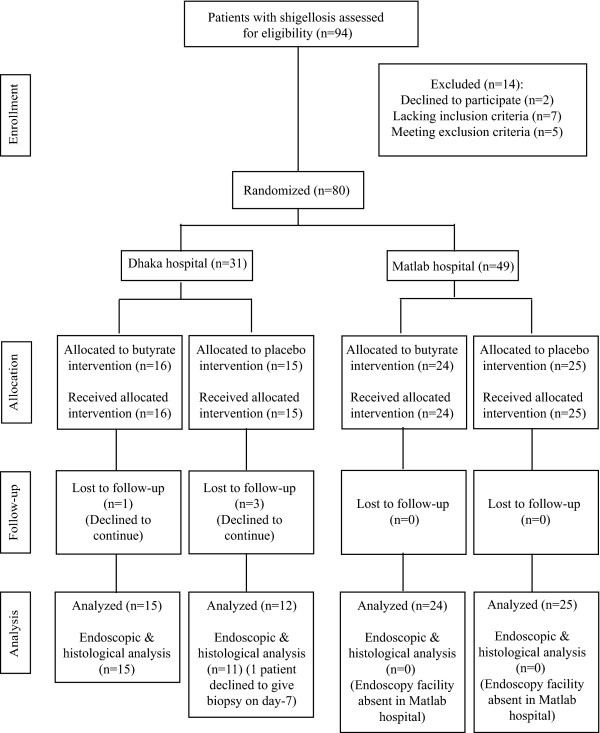
Flow diagram for enrollment, allocation, follow-up and analysis of study patients according to CONSORT.

Figure 1 shows the flow of patients from screening, enrollment, allocation to follow-up and analysis. Forty two male and 34 female patients with a mean age of 34.5 years and average body weight of 45.4 kg were analyzed in the study from January 2005 to January 2009. There were no significant differences in the baseline characteristics and the clinical features between butyrate and placebo treated patients (Table 1).

**Table 1:** **Demographic data of*Shigella*-infected patients on the day of enrollment**^a^

**Features**	**Study patients (N = 76)**		**p value**^**b**^
	**Intervention group**	**Placebo group**	
	**n = 39**	**n = 37**	
Age, yrs	34.72 ± 13.80	34.97 ± 11.20	0.93
Sex, Male: Female	23:16	19:18	0.66
Body weight, kg	46.04 ± 6.08	45.03 ± 6.96	0.5
Duration of diarrhea, days	3.41 ± 1.46	3.21 ± 0.71	0.97
Frequency of defecation, times/d	4.41 ± 4	5.06 ± 4	0.32
Stool output, g	124.71 ±148.35	83.42 ± 77.69	0.21
Fever, n (%)	18 (46.1%)	16 (43.2%)	0.82
Tenesmus, n (%)	25 (64.1%)	26 (70.3%)	0.63
Abdominal cramping, n (%)	32 (82%)	31 (83.8%)	1.0
Blood in stool, n (%)	17 (43.6%)	17 (45.9%)	1.0

^a^Data are given as mean ± standard deviation, proportion of patients or number of patients (% of total patients in each group).

^b^Student-t test or Chi-square test was used to compare between the groups for quantitative or categorical data, respectively. Significance: p≤ 0.05.

### Effect of butyrate treatment on clinical and microbiological outcomes in shigellosis

There were no significant differences in the clinical recovery between the Intervention and Placebo groups of patients in terms of disease scores (p = 0.73) (Data not shown). However, routine microscopic examination of stool samples revealed significant reduction in pus cells (p = 0.009) and macrophages (p = 0.043) by day 2 in the Intervention group compared with the Placebo group (Table 2). Since all patients received antibiotic treatment, *Shigella* bacterial count in the stool samples disappeared within 48 hours in all patients (data not shown) and hence, the effects of butyrate therapy on bacterial counts could not be evaluated.

**Table 2:** **Comparison of stool microscopic outcomes between Intervention and Placebo groups of patients with shigellosis**^a^

	**No. of patient improved n (%) compared to day 1**		**Odds ratio**^**b**^**(95%CI)**	**p value**^**b**^
**Microscopic features in stool**	**Intervention group n = 39**	**Placebo group n = 37**		
RBC at day-2	29 (74.4)	22 (59.5)	1.85 (0.7-4.9)	0.22
RBC at day-3	32 (82))	31 (83.8)	0.88 (0.3-2.9)	1.0
Pus cell at day-2	30 (76.9)	17 (45.9)	3.72 (1.4-10)	0.009
Pus cell at day-3	32 (82)	30 (81.1)	1.1 (0.3-3.4)	1.0
Macrophage at day-2	32 (82)	22 (59.5)	3.12 (1.1-8.9)	0.043
Macrophage at day-3	38 (97.4)	34 (91.9)	3.3 (0.3-33.8)	0.35

**Abbreviations:***RBC* Red blood cell, *CI* Confidence interval.

^a^Data are given as number of patients (% of total patients in each group).

^b^Chi-square test was used to compare between the groups. Significance: p≤ 0.05.

### Butyrate treatment reduces inflammation of rectal mucosa in shigellosis

Sigmoidoscopic examination showed that all 15 patients in the Intervention group had inflammation in the rectal mucosa, either mild (n = 7), moderate (n = 5) or severe (n = 3) on day 1. In the Placebo group, all 11 patients had either mild (n = 6), moderate (n = 4) or severe (n = 1) inflammation on day 1. Rectal inflammation was healed/reduced on day 7 in 11 patients (73.3%) in the Intervention group in contrast to 6 patients (54.5%) in the Placebo group (Table 3). Notably, in the Intervention group, inflammation was completely healed in 9 patients (60%) by day 7, who had presented either severe (n = 3), moderate (n = 2) or mild (n = 4) inflammation on day 1 (Table 3). In the Placebo group, 4 patients (36%) including 1 with moderate and 3 with mild inflammation on day 1 showed no inflammation by day 7 (Table 3).

**Table 3:** **Comparison of rectal inflammation between Intervention and Placebo groups of patients with shigellosis**^a^

**Assessment of Inflammation on day 7**	**Number of patients improved n (%) compared to day 1**		**Odds ratio**^**b**^**(95**% **CI)**	**p value**^**b**^	**Number of patients healed n (%) compared to day 1**		**Odds ratio**^**b**^**(95**% **CI)**	**p value**^**b**^
	**Intervention group**	**Placebo group**			**Intervention group**	**Placebo group**		
By sigmoidoscopy	11 (73.3)	6 (54.5)	2.29	0.324	9 (60)	4 (36)	2.63	0.239
	n = 15	n = 11	(0.4-11.9)		n = 15	n = 11	(0.5-13.1)	
By histology	13 (92.8)	5 (50)	13	0.035	11 (78.6)	1(10)	33	0.005
	n = 14^c^	n = 10^c^	(1.2-140.7)		n = 14^c^	n = 10^c^	(2.9-374.3)	

**Abbreviation:***CI* Confidence interval.

^a^Data are given as number of patients (% of total patients in each group).

^b^Chi-square test was used to compare between groups. Significance: p≤ 0.05.

^c^One patient in the Intervention and one in the Placebo group did not have histological feature of inflammation on day 1 and thus excluded from the analysis.

**Figure 2:**
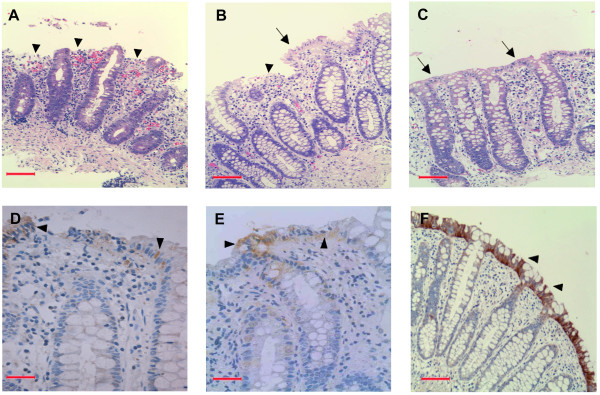
**Rectal tissues from adult patients with shigellosis representing histology (A-C) and LL-37 immunostaining (D-F). (A)** SE was completely eroded at the onset of disease (arrow heads). Huge appearance of blood (deep pink) due to hemorrhage was also evident in LP. **(B)** Partial recovery (arrow) of epithelial erosion (arrow head) and hemorrhage on day 7 following placebo treatments. **(C)** The epithelial erosion (arrows) and hemorrhage were completely healed by day 7 after treatment with butyrate. **(D)** Low expression of LL-37 in the SE (arrow heads) on day 1. **(E)** LL-37 expression in SE was still low (arrow heads) on day 7 after placebo treatment. **(F)** Treatment with butyrate led to higher and intense expression of LL-37 in SE (arrow heads) on day 7. SE: surface epithelium; LP: lamina propria. In figures A, B, C and F, bars equal to 100 μm. In figures D and E, bars equal to 50 μm.

Histological analysis revealed that 14 patients in the Intervention group had inflammation in the rectal mucosa on day 1; either mild (n = 7), moderate (n = 4) or severe (n = 3) (Figure 2). Ten patients in the Placebo group had either mild (n = 5), moderate (n = 3) or severe (n = 2) inflammation. One patient in each group had no inflammation on day 1 and was thus excluded from analysis. On day 7, 13 patients (92.8%) in the Intervention group had improved histological features (Figure 2) of inflammation in contrast to 5 patients (50%) in the Placebo group (Table 3). The number of patients with improved histology on day 7 was significantly higher in the Intervention group than that in the Placebo group (p = 0.035) (Table 3). In the Intervention group, histology was normal (Figure 2) by day 7 in 11 patients (78.6%) (1 patient with severe, 3 patients with moderate and 7 patients with mild inflammation on day 1) (Table 3). In the Placebo group, only 1 patient (10%) had completely healed rectal mucosa on day 7, which was significantly lower compared with the Intervention group (p = 0.005) (Table 3). Notably, 1 patient with moderate inflammation on day 1 got worse with severe histology on day 7 in the Placebo group. These data suggested the contribution of butyrate treatment in the reduction of rectal inflammation.

### Effect of butyrate treatment on release of pro-inflammatory cytokines in stool

**Figure 3:**
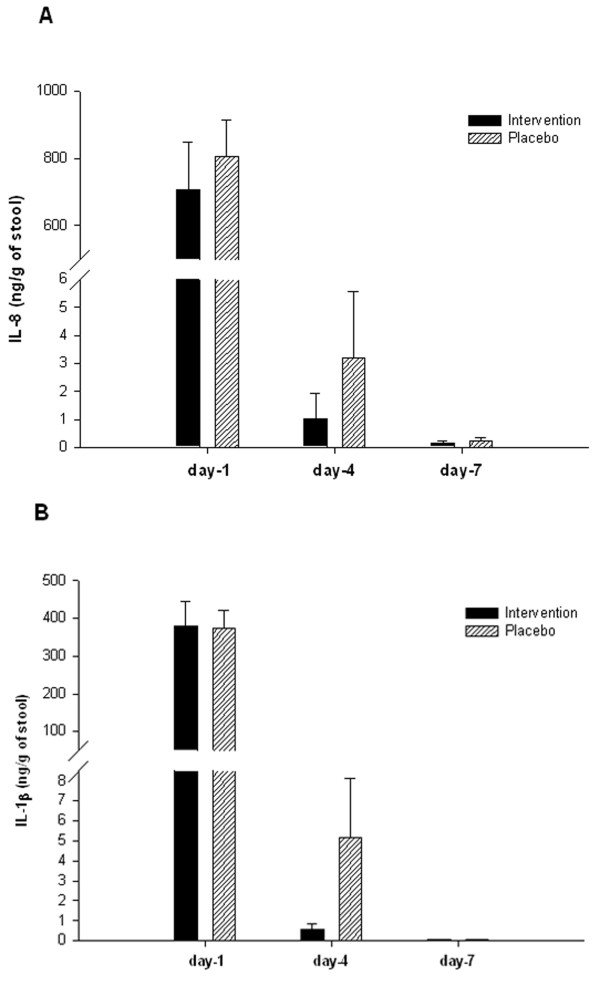
**Levels of Pro-inflammatory cytokines in the stool of*Shigella*-infected patients, treated with butyrate or placebo.** Stool specimens were collected on indicated time points from Intervention (n = 39) and Placebo (n = 37) groups of patients. Concentrations of **(A)** IL-8 and **(B)** IL-1β in stool extracts were measured by enzyme linked immunosorbent assay (ELISA). Data are represented as mean ± SEM. Two-way repeated measure ANOVA was performed to determine significant interaction between butyrate and placebo therapy on different days, and when interaction was significant the Holm-Sidak post hoc comparison procedure was used to compare the effects of butyrate therapy on outcome measures. Significance: p≤ 0.05. Levels of IL-8 and IL-1β diminished significantly from day 1 to days 4 and 7 (≤ 0.001) in both groups. Diminution of IL-8 in the Intervention group was significantly higher than the Placebo group (p = 0.048). Attenuation of IL-1β in the Intervention group was higher but not significant compared with the Placebo group (p = 0.078). IL-8: interleukin-8; IL-1β: interleukin-1β.

Concentration of IL-8 in the stool declined significantly over time (from day 1 to days 4 and 7) (p ≤ 0.001) in both Placebo and Intervention groups. However, the decline in the Intervention group was significantly higher compared with that found in the Placebo group (p = 0.048) (Figure 3A). Similarly, IL-1β level in the stool reduced in both groups over time, and the reduction was higher in the Intervention group compared with the Placebo group, although the difference was not significant (p = 0.078) (Figure 3B). In terms of the decrease of IL-8 level in the stool on day 4, the effect size was 0.18 (small) for the Intervention group compared with the Placebo group.

### Effect of butyrate treatment on LL-37 expression in rectal mucosa

**Figure 4:**
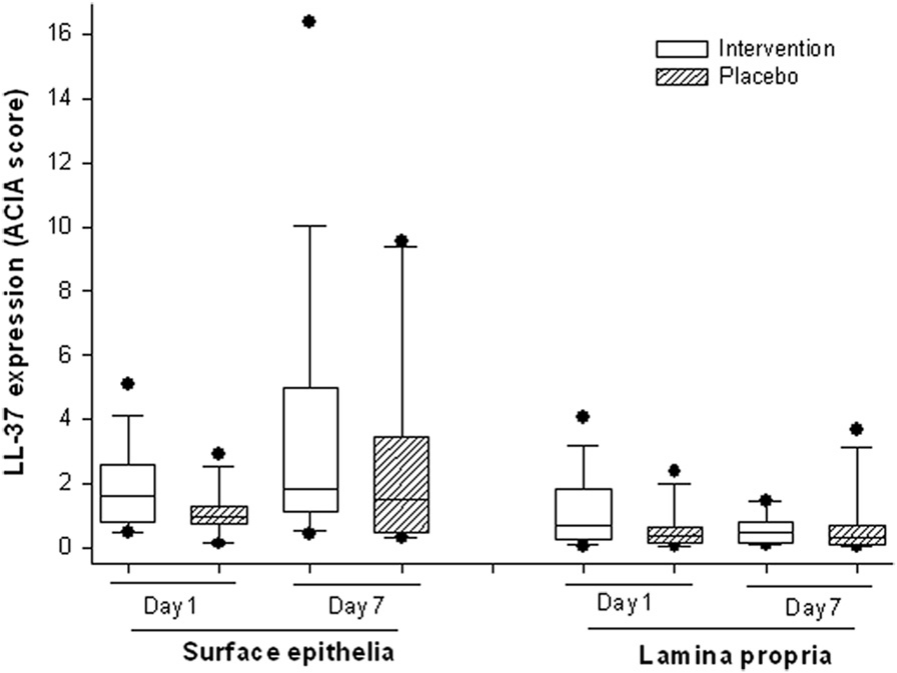
**LL-37 expression in the rectal mucosa of Intervention or Placebo groups of patients with shigellosis.** Rectal biopsies were obtained on day 1 and day 7 from a subgroup of *Shigella*-infected patients, treated with butyrate (n = 15) or placebo (n = 11). Immunohistochemical detection of LL-37 was performed in paraffin sections. SE and LP were separately assessed for the quantification of LL-37 staining in each tissue section and the results were given as ACIA (Acquired Computerized Image Analysis) score. Lower and upper boundaries of the boxes and the horizontal bars in between indicate 25^th^ percentile, 75th percentile and group median respectively. Single lines extending from the boxes represent lower and upper quartiles. Each circle above or below the boxes indicates one outlier. Two-way repeated measure ANOVA was performed to determine significant interaction between butyrate and placebo therapy on different days, and when interaction was significant the Holm-Sidak post hoc comparison procedure was used to compare the effects of butyrate therapy on outcome measures. Significance: p≤ 0.05. Expression of LL-37 in SE increased significantly from day 1 to day 7 in the Intervention group compared with the Placebo group (p = 0.04). There was no significant changes over time between groups in the expression of LL-37 in LP (p = 0.14). SE- surface epithelium; LP-lamina propria.

There was a significant increase in the expression of LL-37 in the surface epithelium (SE) of the rectum from day 1 to day 7 in the Intervention group compared with the Placebo group (p = 0.04) (Figures 2 and 4). No significant changes over time were observed between the two groups in the expression of LL-37 in the lamina propria (LP) (p = 0.14) (Figure 4). The effect size for increased epithelial LL-37 expression on day 7 was small (0.21) for the Intervention group compared with the Placebo group.

### Effect of butyrate treatment on release of antimicrobial peptides in stool

**Figure 5:**
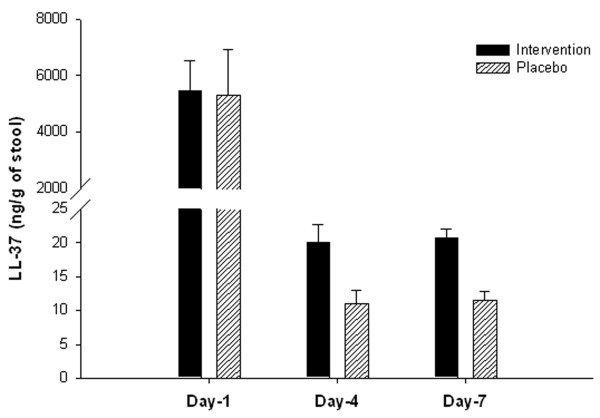
**LL-37 level in the stool of*Shigella*-infected patients, treated with butyrate or placebo.** Stool specimens were collected on indicated time points from Intervention (n = 39) or Placebo (n = 37) groups of patients. Level of LL-37 in stool extracts was measured by enzyme linked immunosorbent assay (ELISA). Data are represented as mean ± SEM. Two-way repeated measure ANOVA was performed to determine significant interaction between butyrate and placebo therapy on different days, and when interaction was significant the Holm-Sidak post hoc comparison procedure was used to compare the effects of butyrate therapy on outcome measures. Significance: p≤ 0.05. Concentration of LL-37 decreased significantly from day 1 to days 4 and 7 (< 0.001) in both groups. The decrease was significantly higher in the Placebo group than that in the Intervention group (p <0.001).

A significant decrease in LL-37 level in the stool from day 1 to days 4 and 7 was observed (p <0.001) for both Placebo and Intervention groups. However, LL-37 concentration on day 4 and day 7 in the Intervention group remained significantly higher than those in the Placebo group (p <0.001) (Figure 5). There was no significant difference between the two groups in terms of the levels of HBD-1 and HBD-3 throughout the study period (data not shown). For elevated concentration of LL-37 in the stool on days 4 and 7, the effect size was 0.96 (large) and 1.53 (large), respectively for the Intervention group compared with the Placebo group.

### Biosafety of topical butyrate treatment

The serum levels of urea, alanine transaminase and γ-glutamyl transferase were within the normal range in both Intervention and Placebo groups of patients (Table 4). The levels of creatinine was slightly below the normal range. However, there was no difference in the creatinine levels between the Intervention and the Placebo groups of patients (Table 4).

**Table 4:** **Levels of hepatic and renal biomarkers in serum of*Shigella*-infected patients at different time**^**a**^

**Patient groups**	**Day**	**Renal biomarkers**		**Hepatic Biomarkers**	
		**Creatinine (mg/dL)**^**b**^	**Urea (mg/dL)**^**b**^	**ALT (U/L)**^**b**^	**γ-GT (U/L)**^**b**^
Intervention	1	0.51 ± 0.04	21.3 ± 3	15.5 ± 1.6	11.4 ± 0.8
	4	0.43 ± 0.02	18 ± 1.1	21.9 ± 2.3	11 ± 0.6
Placebo	1	0.49 ± 0.03	18.7 ± 2.6	18.6 ± 2.2	13.4 ± 2.7
	4	0.40 ± 0.02	17.3 ± 1.5	21.1 ± 2.4	18.9 ± 4

**Abbreviation:***ALT* Alanine transaminase, *γ−GT* Gamma glutamyl transferase.

^a^Data are given as mean ± standard error of mean.

^b^Normal reference values of creatinine: 0.6-1.2 mg/dL (men) and 0.5-1.1 mg/dL (women); urea: 6-24 mg/dL; ALT: 0.01-41U/L (men) and 0.01-31.0U/L (women) and γ−GT 9-40U/L (men) and 0-35U/L (women).

## Discussion

The results of this study show that adjunct therapy with sodium butyrate enema leads to an early decline in frequency of inflammatory cells and concentrations of pro-inflammatory cytokines in the stool, but has no obvious effect on clinical recovery from shigellosis. In a subgroup of *Shigella*-infected patients, butyrate treatment resulted in early improvement of rectal inflammation compared with placebo-treated patients. Furthermore, the expression of cathelicidin LL-37 in the rectal epithelium was significantly enhanced following butyrate treatment.

In our earlier study in a rabbit model of shigellosis, butyrate treatment resulted in a marked improvement in clinical outcomes and early reduction of *Shigella* count in the stool [11]. We did not observe similar effects in this study. It is worth mentioning that while rabbits were given butyrate treatment without any antibiotics, patients were given butyrate in addition to antibiotics. Therefore, it is not unexpected that the clinical features and *Shigella* count in the stool subsided in all study patients simultaneously.

The potential anti-inflammatory effects of butyrate have long been documented. Preventive and therapeutic effects of butyrate on inflammation were evaluated in animal models of colitis [16-18]. In patients with ulcerative colitis (UC) or Crohn’s disease (CD), oral/topical administration of butyrate (or SCFA mixture), or stimulation of luminal butyrate production by feeding dietary fibers, have been shown to reduce clinical and inflammatory index in several studies (reviewed in [19,20]). In experimental shigellosis in rabbits, the inflammatory parameters were also reduced with colonic infusion of an SCFA mixture [10], or by oral administration of butyrate [11]. No adverse effects of butyrate treatment were reported in these studies. Here, we have shown that local luminal administration of butyrate can significantly improve the histological features of inflammation in the rectum in a subgroup of patients. Butyrate treatment also resulted in an early decline in pus cells, macrophages and pro-inflammatory cytokines in the stool. Hence, butyrate treatment can resolve the persistence of inflammation, which often occurs after clinical recovery from shigellosis following antibiotic treatment [3,21]. Furthermore, patients did not exhibit untoward effects of butyrate treatment as assessed by the levels of renal and hepatic biomarkers.

The anti-inflammatory effect of butyrate is mediated primarily through the inhibition of nuclear factor κB (NF-κB) activation in the large intestinal mucosa [22-24]. Dysregulated activation of NF-κB in animal models of colitis, and in patients with UC or CD, was reported to be inhibited by butyrate, which correlated with decreased inflammation [25,26]. The anti-inflammatory activity of butyrate might also be due to the activation of peroxisome proliferator-activated receptor-γ (PPARγ), a ligand-activated transcription factor in colonic epithelial cells [27,28], and the inhibition of interferon-γ signaling [29]. Moreover, involvement of the G-protein coupled receptor (GPCR) in the anti-inflammatory effect of SCFA has been demonstrated recently in animal models of colitis, arthritis and asthma [30].

Treatment with butyrate showed increased expression of LL-37 in the rectal epithelia compared with placebo treatment, although there was a general increase in its expression in both groups after one week. This was in accordance with our previous finding in experimental shigellosis, where butyrate treatment counteracted the downregulation of rabbit cathelicidin in the rectal epithelia [11]. However, the effect size in patients was small, which could be due to the fact that butyrate was given as an enema in patients in contrast to oral therapy in the rabbit model. Patients may not have completely retained the butyrate after infusion because of repeated defecation, which would affect the absorption of butyrate in serum. Recently, we have shown that the systemic dissemination of butyrate is necessary to induce cathelicidin expression in epithelial cells [31]. In parallel to epithelial expression, the release of LL-37 peptide in the stool also remained significantly higher on days 4 and 7 in the Intervention group. Since there was significant reduction of inflammatory cells in stool from the Intervention group, the prolonged secretion of LL-37 in the stool may have originated from the healed epithelium of the large intestine and may play a role in bactericidal activities.

The current study has a number of limitations. Since both groups of patients were given antibiotics, it was not possible to evaluate whether butyrate treatment enhanced shigellacidal activity in the stool. Butyrate was given as an enema instead of oral therapy as in rabbits since the bad smell of butyrate makes it unsuitable for oral therapy in humans. Repeated use of enemas is troublesome, especially with regards to patient compliance and the need for hospital facilities. Oral administration of enteric coated tablets containing butyrate can be a better alternative, which was proved to be effective previously in patients with ulcerative colitis [32]. Induction of luminal butyrate via ingestion of fermentable fiber supplementation, which has been successfully used in clinical trials for ulcerative colitis (reviewed in [20]) and childhood shigellosis [12], may also be suitable for healing inflammation in shigellosis. In addition, consumption of probiotics has recently been suggested as an interesting approach to reduce intestinal inflammation through the upregulation of luminal levels of butyrate and butyrate-producing commensal bacteria, and the lowering of cecal pH [33]. In fact, these and additional metabolic shifts in T-bet^−/−^Rag2^−/−^ mice were shown to improve colitis scores by creating an unfavorable environment for the colitogenic *Enterobacteriaceae*. However, whether these therapies would also be suitable for the induction of antimicrobial peptides in the epithelium remains to be seen.

## Conclusion

The current study demonstrates that adjunct therapy with butyrate enema during shigellosis promotes healing of the rectal mucosa and reduces luminal content of inflammatory cells and pro-inflammatory cytokines. Butyrate treatment also resulted in enhanced expression of LL-37 in the rectal epithelia and prolonged secretion of LL-37 in the stool. However, efficacy of butyrate in clinical recovery from shigellosis was not evident. Recently, we have shown that sodium 4-phenylbutyrate (PB), a derivative of butyrate without the foul smell, provides similar treatment efficacy as butyrate when given orally to rabbits with experimental shigellosis [31]. Since PB is already an approved drug for treating urea cycle disorder, it holds much promise as a therapeutic alternative for human shigellosis.

## Abbreviations

CONSORT, Consolidated Standards of Reporting Trials; HBD-1, Human beta defensin 1; HBD-3, Human beta defensin 3; IL-1β, Interleukin-1β; IL-8, Interleukin-8; AMP, Antimicrobial peptide; SCFA, Short chain fatty acid; RBC, Red blood cell; RME, Routine microscopic examination; TFA, Trifluoroacetic acid; CFU, Colony forming unit; ELISA, Enzyme linked immunosorbent assay; TBS, Tris-buffer saline; 4-MUP, 4-methylumbelliferyl phosphate; H2O2, Hydrogen per-oxide; ACIA, Acquired Computerized Image Analysis; SE, Surface epithelium; LP, Lamina propria; ES, Effect size; UC, Ulcerative colitis; CD, Crohn’s disease; NF-κB, Nuclear factor κB; PPARγ, Peroxisome proliferator-activated receptor-γ; GPCR, G-protein coupled receptor; CI, Confidence interval.

## Competing interest

The authors declare that they have no competing interest.

## Authors’ contributions

RR, BA, GHG, NHA and JA conceived and designed the trial. NHA performed sigmoidoscopy and collected biopsies. ASMA were responsible for data acquisition and specimen collection. PS, AM and RSR performed the laboratory experiments. RR, PS, AM and RSR carried out the statistical analysis. RR and BA supplied reagents/materials/analysis tools. RR, PS and BA drafted the manuscript. JA, GHG and AC revised the manuscript. All authors approved the final version of the manuscript before submission.

## Pre-publication history

The pre-publication history for this paper can be accessed here:

http://www.biomedcentral.com/1471-2334/12/111/prepub

## References

[B1] Bardhan P Faruque AS Naheed A Sack DA Decrease in shigellosis-related deaths withoutShigellaspp.-specific interventions, Asia Emerg Infect Dis 2010 16 11 1718 1723 10.3201/eid1611.090934 21029529 PMC3294502

[B2] Sansonetti PJ Microbes and microbial toxins: paradigms for microbial-mucosal interactions III. Shigellosis: from symptoms to molecular pathogenesis Am J Physiol Gastrointest Liver Physiol 2001 280 3 G319 G323 11171613 10.1152/ajpgi.2001.280.3.G319

[B3] Raqib R Lindberg AA Wretlind B Bardhan PK Andersson U Andersson J Persistence of local cytokine production in shigellosis in acute and convalescent stages Infect Immun 1995 63 1 289 296 7806368 10.1128/iai.63.1.289-296.1995 PMC172990

[B4] Raqib R Moly PK Sarker P Qadri F Alam NH Mathan M Andersson J Persistence of mucosal mast cells and eosinophils in Shigella-infected children Infect Immun 2003 71 5 2684 2692 10.1128/IAI.71.5.2684-2692.2003 12704143 PMC153256

[B5] Niyogi SK Shigellosis J Microbiol 2005 43 2 133 143 15880088

[B6] Zasloff M Antimicrobial peptides of multicellular organisms Nature 2002 415 6870 389 395 10.1038/415389a 11807545

[B7] Islam D Bandholtz L Nilsson J Wigzell H Christensson B Agerberth B Gudmundsson G Downregulation of bactericidal peptides in enteric infections: a novel immune escape mechanism with bacterial DNA as a potential regulator Nat Med 2001 7 2 180 185 10.1038/84627 11175848

[B8] Sperandio B Regnault B Guo J Zhang Z Stanley SL Sansonetti PJ Pedron T Virulent Shigella flexneri subverts the host innate immune response through manipulation of antimicrobial peptide gene expression J Exp Med 2008 205 5 1121 1132 10.1084/jem.20071698 18426984 PMC2373844

[B9] Mortensen PB Clausen MR Short-chain fatty acids in the human colon: relation to gastrointestinal health and disease Scand J Gastroenterol Suppl 1996 216 132 148 8726286 10.3109/00365529609094568

[B10] Rabbani GH Albert MJ Hamidur Rahman AS Moyenul Isalm M Nasirul Islam KM Alam K Short-chain fatty acids improve clinical, pathologic, and microbiologic features of experimental shigellosis J Infect Dis 1999 179 2 390 397 10.1086/314584 9878023

[B11] Raqib R Sarker P Bergman P Ara G Lindh M Sack DA Nasirul Islam KM Gudmundsson GH Andersson J Agerberth B Improved outcome in shigellosis associated with butyrate induction of an endogenous peptide antibiotic Proc Natl Acad Sci U S A 2006 103 24 9178 9183 10.1073/pnas.0602888103 16740661 PMC1482586

[B12] Rabbani GH Ahmed S Hossain I Islam R Marni F Akhtar M Majid N Green banana reduces clinical severity of childhood shigellosis: a double-blind, randomized, controlled clinical trial Pediatr Infect Dis J 2009 28 5 420 425 10.1097/INF.0b013e31819510b5 19319017

[B13] Jakobovits SL Travis SP Management of acute severe colitis Br Med Bull 2005 75-76 131 144 16847166 10.1093/bmb/ldl001

[B14] Raqib R Reinholt FP Bardhan PK Karnell A Lindberg AA Immunopathological patterns in the rectal mucosa of patients with shigellosis: expression of HLA-DR antigens and T-lymphocyte subsets APMIS 1994 102 5 371 380 8024739 10.1111/j.1699-0463.1994.tb04886.x

[B15] Cunnane G Bjork L Ulfgren AK Lindblad S FitzGerald O Bresnihan B Andersson U Quantitative analysis of synovial membrane inflammation: a comparison between automated and conventional microscopic measurements Ann Rheum Dis 1999 58 8 493 499 10.1136/ard.58.8.493 10419868 PMC1752933

[B16] Andoh A Bamba T Sasaki M Physiological and anti-inflammatory roles of dietary fiber and butyrate in intestinal functions JPEN J Parenter Enteral Nutr 1999 23 5 Suppl S70 S73 10.1177/014860719902300518 10483900

[B17] Butzner JD Parmar R Bell CJ Dalal V Butyrate enema therapy stimulates mucosal repair in experimental colitis in the rat Gut 1996 38 4 568 573 10.1136/gut.38.4.568 8707089 PMC1383116

[B18] Song M Xia B Li J Effects of topical treatment of sodium butyrate and 5-aminosalicylic acid on expression of trefoil factor 3, interleukin 1β, and nuclear factor κB in trinitrobenzene sulphonic acid induced colitis in rats Postgrad Med J 2006 82 964 130 135 10.1136/pgmj.2005.037945 16461476 PMC2596699

[B19] Canani RB Costanzo MD Leone L Pedata M Meli R Calignano A Potential beneficial effects of butyrate in intestinal and extraintestinal diseases World J Gastroenterol 2011 17 12 1519 1528 10.3748/wjg.v17.i12.1519 21472114 PMC3070119

[B20] Hamer HM Jonkers D Venema K Vanhoutvin S Troost FJ Brummer RJ Review article: the role of butyrate on colonic function Aliment Pharmacol Ther 2008 27 2 104 119 17973645 10.1111/j.1365-2036.2007.03562.x

[B21] Raqib R Wretlind B Andersson J Lindberg AA Cytokine secretion in acute shigellosis is correlated to disease activity and directed more to stool than to plasma J Infect Dis 1995 171 2 376 384 10.1093/infdis/171.2.376 7531208

[B22] Andoh A Fujiyama Y Hata K Araki Y Takaya H Shimada M Bamba T Counter-regulatory effect of sodium butyrate on tumour necrosis factor-alpha (TNF-α)-induced complement C3 and factor B biosynthesis in human intestinal epithelial cells Clin Exp Immunol 1999 118 1 23 29 10.1046/j.1365-2249.1999.01038.x 10540155 PMC1905403

[B23] Inan MS Rasoulpour RJ Yin L Hubbard AK Rosenberg DW Giardina C The luminal short-chain fatty acid butyrate modulates NF-κB activity in a human colonic epithelial cell line Gastroenterology 2000 118 4 724 734 10.1016/S0016-5085(00)70142-9 10734024

[B24] Place RF Noonan EJ Giardina C HDAC inhibition prevents NF-κB activation by suppressing proteasome activity: down-regulation of proteasome subunit expression stabilizes IκBα Biochem Pharmacol 2005 70 3 394 406 10.1016/j.bcp.2005.04.030 15950952

[B25] Luhrs H Gerke T Muller JG Melcher R Schauber J Boxberge F Scheppach W Menzel T Butyrate inhibits NF-κB activation in lamina propria macrophages of patients with ulcerative colitis Scand J Gastroenterol 2002 37 4 458 466 10.1080/003655202317316105 11989838

[B26] Segain JP Raingeard de la Bletiere D Bourreille A Leray V Gervois N Rosales C Ferrier L Bonnet C Blottiere HM Galmiche JP Butyrate inhibits inflammatory responses through NFκB inhibition: implications for Crohn's disease Gut 2000 47 3 397 403 10.1136/gut.47.3.397 10940278 PMC1728045

[B27] Kinoshita M Suzuki Y Saito Y Butyrate reduces colonic paracellular permeability by enhancing PPARγ activation Biochem Biophys Res Commun 2002 293 2 827 831 10.1016/S0006-291X(02)00294-2 12054544

[B28] Schwab M Reynders V Loitsch S Steinhilber D Stein J Schroder O Involvement of different nuclear hormone receptors in butyrate-mediated inhibition of inducible NFκB signalling Mol Immunol 2007 44 15 3625 3632 10.1016/j.molimm.2007.04.010 17521736

[B29] Klampfer L Huang J Sasazuki T Shirasawa S Augenlicht L Inhibition of interferon γ signaling by the short chain fatty acid butyrate Mol Cancer Res 2003 1 11 855 862 14517348

[B30] Maslowski KM Vieira AT Ng A Kranich J Sierro F Yu D Schilter HC Rolph MS Mackay F Artis D Regulation of inflammatory responses by gut microbiota and chemoattractant receptor GPR43 Nature 2009 461 7268 1282 1286 10.1038/nature08530 19865172 PMC3256734

[B31] Sarker P Ahmed S Tiash S Rekha RS Stromberg R Andersson J Bergman P Gudmundsson GH Agerberth B Raqib R Phenylbutyrate counteracts Shigella mediated downregulation of cathelicidin in rabbit lung and intestinal epithelia: a potential therapeutic strategy PLoS One 2011 6 6 e20637 10.1371/journal.pone.0020637 21673991 PMC3108617

[B32] Vernia P Monteleone G Grandinetti G Villotti G Di Giulio E Frieri G Marcheggiano A Pallone F Caprilli R Torsoli A Combined oral sodium butyrate and mesalazine treatment compared to oral mesalazine alone in ulcerative colitis: randomized, double-blind, placebo-controlled pilot study Dig Dis Sci 2000 45 5 976 981 10.1023/A:1005537411244 10795763

[B33] Veiga P Gallini CA Beal C Michaud M Delaney ML DuBois A Khlebnikov A van Hylckama Vlieg JE Punit S Glickman JN Bifidobacterium animalis subsp. lactis fermented milk product reduces inflammation by altering a niche for colitogenic microbes Proc Natl Acad Sci U S A 2010 107 42 18132 18137 10.1073/pnas.1011737107 20921388 PMC2964251

